# Sociocultural Influences Contribute to Overeating and Unhealthy Eating: Creating and Maintaining an Obesogenic Social Environment in Indigenous Communities in Urban Fiji

**DOI:** 10.3390/nu14142803

**Published:** 2022-07-08

**Authors:** Shazna M. Buksh, John B. F. de Wit, Phillipa Hay

**Affiliations:** 1 School of Law and Social Sciences, The University of the South Pacific, Laucala Campus, Suva 1168, Fiji; 2 Eating Disorders and Body Image (EDBI), School of Medicine, Western Sydney University, Penrith, NSW 2751, Australia; p.hay@westernsydney.edu.au; 3 Department of Interdisciplinary Social Science, Faculty of Social and Behavioural Science, Utrecht University, 3584 CS Utrecht, The Netherlands; j.dewit@uu.nl; 4 Translational Health Research Institute, Western Sydney University, Penrith, NSW 2751, Australia

**Keywords:** obesity, sociocultural factors, unhealthy eating, overeating, nutrition transition, fast food, junk food, processed meat

## Abstract

Pacific Island countries (PICs), such as Fiji, lead the world statistics in obesity and deaths caused by non-communicable diseases. The impacts of obesity overburden the healthcare system and social services and have major impacts on the Fijian economy. This study is the first of its kind to undertake an in-depth exploration of the determinants of the obesity epidemic in PICs by exploring the sociocultural influences and situations that impact nutrient transitions, overconsumption, and unhealthy eating in an urban indigenous community. In-depth qualitative interviews were conducted with 15 indigenous Fijian women from the largest urban center in Fiji who self-identified as gatekeepers of family meal planning, preparing, and shopping for groceries, fruits, and vegetables. The women identified several cultural norms and expectations of both the individuals providing the food and the individuals consuming the food that create and maintain an obesogenic social environment. Moreover, participants also shared a misplaced value on meat, energy-dense foods, junk food, and fast foods that further contribute to unhealthy eating and overeating within this urban indigenous community. These novel findings highlight the importance of considering sociocultural influences on unhealthy eating and overeating and may be used to assist decision-makers in developing contextualized obesity prevention strategies and health messaging to target obesity in this community.

## 1. Introduction

Global obesity rates have tripled since 1975 [1], and Pacific Island countries (PICs) undoubtedly share the greatest part of the obesity burden: of the top 20 countries with the highest percentages of adult obesity, 14 are PICs [2,3]. The high rates of obesity have also dramatically increased the rates of non-communicable diseases (NCDs) in small PICs such as Fiji, which has one of the highest rates of death caused by NCDs in the world. In Fiji, 84% of all deaths are caused by NCDs, and nearly a third of Fijian adults are at risk of dying prematurely due to NCDs [4]. The high rates of NCDs and premature death not only overburden the healthcare system and social services but also have devastating impacts on an already struggling and developing economy [5]. Moreover, there is mounting evidence for a relationship between the obesity epidemic and the COVID-19 pandemic, especially in relation to the (1) adoption of poor dietary practices and increases in body mass index (BMI) during COVID-19 lockdowns (e.g., [6,7]) and (2) the heightened severity of the disease amongst obese young adults (e.g., [8,9]). Therefore, the current COVID-19 pandemic further exacerbates the population health impact of obesity.

Fox, Feng and Asal [10] identified two theories to explain the growing obesity epidemic in low- and middle-income countries (LMIC). World-systems theory, also known as dependency theory, largely attributes the obesity epidemic to dietary transitions caused by globalization. Economic globalization, especially trade liberalization, increases the importation of, and therefore access to, ultra-processed foods high in sugar and unhealthy fats in LMIC. In addition, cultural globalization increases the appeal of western lifestyles, including the desirability of associated foods [10] (p. 1). In contrast, modernization theory attributes the rise in obesity in LMIC mostly to domestic factors. According to this theory, economic growth increases urbanization and the participation of women in the labor force, which contributes to a shift from traditional plant-based diets to diets consisting of foods that are convenient and affordable, such as ultra-processed foods and meat [10] (p. 2).

The dietary transitions observed in PICs show some level of support for both theories. Dietary transitions in PICs have been largely attributed to a change from subsistence economies to cash economies, an increase in urbanization, an increase in the importation of ultra-processed foods, and economic growth due to an increase in investments and the injection of international aid in the economies of PICs [11]. Explanations based on dependency theory have, however, long garnered the most attention, as dietary transitions were mainly recorded post-colonization and after World Trade Organization agreements allowing for free trade policies came into effect (e.g., [12]). For example, trade policies and their impacts on processed food consumption dominated early literature on dietary transition in PICs and continues to be a key focus of research in this area. Studies found that trade liberalization, in particular, resulted not only in greater imports of ultra-processed foods but also that the ensuing competition caused a reduction in the prices of domestically-produced processed foods in low- to middle-income PICs, ultimately making processed food more affordable within Pacific communities (e.g., [13,14,15,16]). Nutrition reports from PICs also confirm an increase in the consumption of processed foods, including refined cereals, sugar-sweetened beverages, meat, and fats, after trade polices (including trade liberalization) came into effect in PICs (e.g., [12,14,15,17]). Consequently, most of the recommendations on food-related interventions in PICs focus on a re-assessment of trade policies, such as increasing taxation on processed foods and limiting imports of unhealthy processed foods, including meat (e.g., [17,18,19]).

More recently, the focus of studies looking at explanations for dietary transitions in PICs and their impact on health have also started examining domestic factors. Several inter-related and overlapping factors have been identified. Increases in urbanization in PIC communities, more women joining the labor force, and income growth have increased the affordability of ultra-processed foods, meat, and fast-foods [10,12,15,20,21,22,23]. Moreover, the convenience of these foods (such as in reducing meal preparation time, longer shelf-life, and easy storage) have, in some respects, promoted a transition from a traditional plant-based diet to a diet high in ultra-processed foods and fast foods, especially in urban communities and households with both working parents (e.g., [23]). Another salient factor, related to cultural globalization, which may be contributing to the nutrition transition in PICs is the desire for western-type meals and fast foods [6,12,23]. Seubsman, Kelly, Yuthapornpinit, and Sleigh [24] (p. 672) suggest that one of the reasons why fast foods are “valued” in LMIC is because they are equated to having a modern lifestyle and, therefore, fast-food restaurants are chosen venues for celebrations and social events, which further reinforces the desirability of these foods. In combination, these studies have identified individual factors (e.g., gender, age, ethnicity, income, convenience, and taste); physical environment factors (e.g., access to processed foods, fast foods, fruits, and vegetables), and macro-economic factors (e.g., trade policies) that impact eating. However, very little research has been conducted on how the social environment might impact the high overweight and obesity rates observed amongst Pacific islanders. Moreover, the ways in which sociocultural factors contribute to obesity risk amongst Pacific islanders are poorly understood.

This study aims to expand the discussion on domestic factors shaping the nutrition transitions that are driving the obesity epidemics in PICs. Specifically, the study aims to address the gap in knowledge of the impact of sociocultural factors by exploring the sociocultural influences and situations that impact food consumption and, especially, overconsumption and unhealthy eating. In Fiji, iTaukei (indigenous Fijians) have significantly higher rates of obesity and higher risk factors for developing NCDs [25]. Furthermore, Fijian urban populations not only have greater access to cheap ultra-processed foods and fast foods but also have higher consumption of these foods [22,26]. An examination of some of the sociocultural influences affecting overeating and unhealthy eating can increase our understanding of the reasons why urban iTaukei communities experience higher rates of obesity and NCD risk factors. Therefore, this study is undertaken with iTaukei women living in the largest urban center in Fiji, the Greater Suva Urban Area (GSUA), which encompasses Suva city, the capital of Fiji, and three smaller towns (Lami, Nausori and Nasinu). The GSUA is home to approximately a third of Fiji’s population and 57% of Fiji’s urban population [27]. Mothers were specifically recruited for this study because, in PICs such as Fiji, mothers are often food gatekeepers, playing key roles in determining food selection, food preparation, and how much is consumed by family members [28]. We anticipated that the findings of this study may assist decision-makers with identifying obesity prevention strategies beyond trade policies and inform the development of contextualized intervention strategies, including health messaging, to target obesity within this community.

## 2. Materials and Methods

### 2.1. Research Design

Inductive thematic analysis was used as a framework for data collection and analysis that were treated as “recursive” processes ([29], p. 86). The study employed semi-structured interviews for an in-depth exploration of sociocultural factors that contribute to overeating and unhealthy eating behaviors in an urban indigenous community in Fiji.

### 2.2. Participants

Fifteen iTaukei mothers who self-identified as Christians participated in the study. Christianity is the dominant religion within iTaukei and 99.2% of iTaukei people ascribe to some denomination of the faith [30]. The women were aged 23–48 years (M = 36.33, SD = 8.18), of whom twelve were married, one was divorced, and two were single mothers. Most of the women lived in suburbs around Suva, Lami, Nasinu, and Nausori, and two of the women lived in villages near Suva and Lami. The women had different occupational backgrounds, including women employed full-time (*n* = 5), full-time or part-time students (*n* = 3), currently unemployed women (*n* = 4), and stay-at-home mothers (*n* = 3). All women had, at least, secondary education.

The mothers self-identified as gatekeepers of family meal planning, preparing, and shopping for groceries, fruits, and vegetables. Family sizes ranged from three to ten people and seven of the women lived in extended family settings; eight women lived with their nuclear families. The number of social gatherings, funeral functions, church meetings, family functions (e.g., weddings, anniversaries, birthdays), and work-related events where food was being served attended by the mothers ranged from once a month to five times a week. Some working women and women who were active members of their churches attended social events two to five times a week.

### 2.3. Data Collection

Purposive sampling was used to recruit women through community leaders to ensure representation of women from different occupational backgrounds, family systems, and locations within the GSUA. The primary inclusion criterion was that women were iTaukei mothers involved in family meal planning, preparation, and shopping. As the study progressed, theoretical sampling was used to ensure representation of women with different experiences in hosting and attending social gatherings, including family celebrations and communal events, as these were identified as important factors in the understanding of sociocultural factors that impacted food consumption.

A semi-structured interview guide was used, which consisted of open-ended questions exploring the impact of culture on what is appropriate and desirable to eat; what factors determine how much people eat; and when overeating is seen as appropriate or is encouraged. As the study took place during COVID-19 lockdowns in GSUA (May to August 2021), participants were prompted to describe their experiences before COVID-19 safety protocols were enforced by the Fijian Government. A companion study on changes in diet, eating behavior, and food purchasing behavior due to these COVID-19 safety protocols that included thirteen of the participants from this study has been published [6]. Due to social-distancing protocols enforced by the government during the time of this study, in-depth interviews were conducted via telephone or video call technology. Interviews were recorded and transcribed verbatim by the first author. All interviews were conducted in English and lasted approximately 50 min. Participants were provided a reimbursement of FJD20 through online money transfer.

### 2.4. Data Analysis

**Figure 1:**
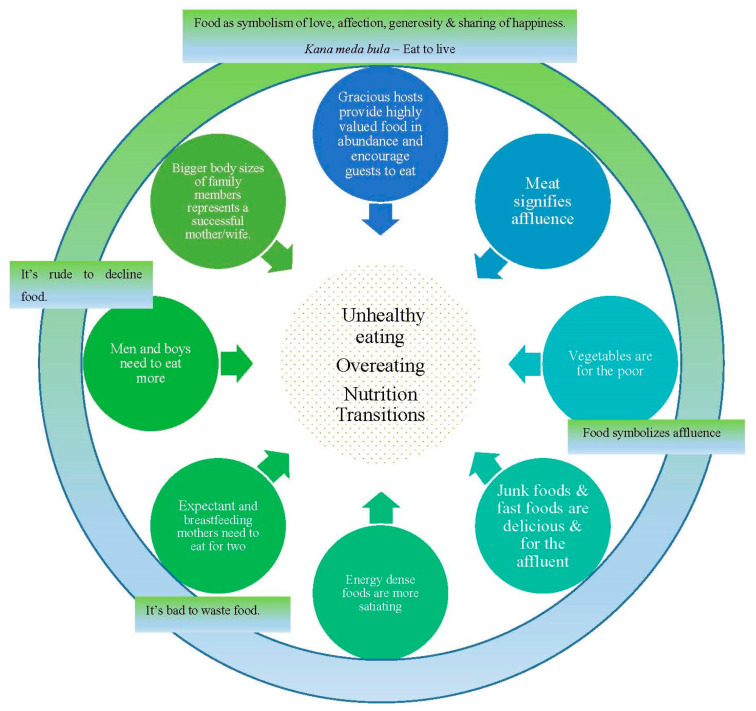
Thematic Map.

Participant recruitment was guided by theoretical saturation of the identified themes. Interviews were stopped when no new information related to the themes was identified after four consecutive interviews [31]. The transcripts were read multiple times to identify initial codes, which were then mapped across all the transcripts. Initial themes that related to the sociocultural influences and situations that impacted food consumption and, especially, overconsumption and unhealthy eating were identified and further refined. A thematic map identifying and illustrating the relationships between the main themes and sub-themes was developed (See Figure 1) and relevant participant quotes were selected to illustrate these themes.

### 2.5. Ethical Approval

Ethical approval for the study was given by the Research and Innovation Office of The University of the South Pacific before the commencement of the study. All participants were given an overview of the study and were informed of their rights to participation, withdrawal, and compensation. All participants gave recorded verbal consent and were allocated pseudonyms to protect their identity.

## 3. Results

The sociocultural factors that may affect food consumption, as shared by the 15 mothers, are presented under two themes, as illustrated in Figure 1: (1) the cultural norms, expectations, and pressures that contribute to overeating and unhealthy eating and (2) the factors that are driving a nutrition transition through the misplaced value on meat, energy dense foods, junk food, and fast foods. Figure 1 also illustrates the overarching norms and beliefs we found that encourage and facilitate overeating and unhealthy eating: Those who provide food must provide valuable food in abundance and encourage eating (as a demonstration of their love, generosity, affluence, and as a way of sharing of happiness) and those who consume the food must accept and finish all of it as it is culturally impolite to decline food and wastage of food is frowned upon. In combination, these sociocultural norms and beliefs contribute to and maintain an obesogenic social environment within this urban indigenous community.

### 3.1. Cultural Norms, Expectations and Pressures That Create and Maintain a Social Environment of Overeating and Unhealthy Eating

The mothers shared the cultural expectation of the individual(s) providing the food, be it the host in social gatherings or the elders of the household, usually women, which was to do their best to provide, in abundance, highly-valued food and/or food that was perceived as “good”. According to the women, the act was demonstrative of love and generosity, a way of sharing happiness, and symbolic of the buying-power of the provider, i.e., their affluence. The person receiving the food, therefore, was not only expected to accept this symbol of love, generosity, and happiness, but also to consume it all. These norms and expectations became more pronounced during social gatherings, and the pressures experienced by the providers and those being offered the food are discussed here.

#### 3.1.1. Generous Hosts Provide Highly-Valued Food in Abundance and Encourage Guests to Eat

All the women who participated in this study shared that food was the main feature of any iTaukei gathering and that a successful gathering is one where there is an abundance of food, especially meat, as the food that is served and offered to guests signifies generosity, happiness, love, affection, and the buying-power of the host. For two reasons, guests were thought to look forward to enjoying the food that might be served. Firstly, guests who generally could not afford to buy beef and pork would get to eat these meats during such gatherings. Secondly, social gatherings are one of the rare occasions where traditional methods of mass cooking, such as the *lovo* (underground earth oven, where food is slow cooked over several hours), are used.

Participants also shared that part of iTaukei hospitality was to encourage the people being served the meal to eat more. Consequently, hosts and mothers routinely encourage guests and family members to overeat. This behavior was reportedly more pronounced during social gatherings or when hosting guests, whereby the host would encourage overeating with reassurances of “plentiful food” or “there is a lot of food left”. Another phrase that participants commonly shared that was used to encourage overeating was “*kana meda bula*”, which directly translates as “eat to live”, but according to the participants it goes beyond just this: It relates to enjoying food during celebrations and in the company of family and friends whilst letting go of any inhibitions that there might be, such as concerns for health, gaining weight, or being already full, as shared by the following participant.

Mel: Kana meda bula! The more you eat, the more you live, in fact the better you live [Laughs]. You leave your, what you say, your diet, or worries about health and enjoy the vibe, the environment and the company [laughs]. Basically, enjoy now and worry about those things later. And so, you get tempted, I mean, who wouldn’t? We love our food and it’s our traditional food and it’s not bad food. So, eat today and I will detox tomorrow [Laughs].

Consequently, participants shared that overconsumption of food during social gatherings was common and encouraged. These expectations and consumption patterns put pressure on the hosts ‘to deliver’, and the resulting host-guest dynamic perpetuated an environment of overconsumption and unhealthy eating during social gatherings.

Mel: As my mother-in-law would say “It’s better for us to have leftovers than to have that pot dry and people can tell that there was less food. If there is still leftovers, then you know your guests ate well and you know, it was a successful function”. And so, we keep refilling the bowls on the serving table and we encourage our guests to eat well. It looks bad if a serving bowl is empty or if run out of food. So, when you cook or cater, you always make sure there’s leftovers.

Maggie: I think that’s just in our culture, the way we meet people during these gatherings, the happiness we feel and one of the way we show love is to offer food.

Leba: Oh, definitely when we have occasions like wedding or something like get-together, cousin said … or family will go [say], “*Kana kana*, c’mon c’mon, we only live once! *Kana meda bula*. It’s been a long [sic] when we meet again come on, this is the only time that we eat!”.

Api: Because when we go to a gathering, the food is in abundance so from my point of view, people just gorge themselves. [] And when people dish a little bit, others will keep encouraging, “c’mon c’mon, there’s more, there’s more. Have some more! There’s plenty of food for everybody, *kana meda bula*” So you are opening that door for people to overeat. [] Also, in parties or social gatherings, you find that people keep nudging each other, sort of encouraging each other to eat more. Like “*mai kana*, *mai kana*”. And it seems to be a cultural thing too. Like part of iTaukei hospitality is to encourage your visitors to eat more. It’s a Pacific culture.

Participants who had hosted a social gathering admitted to over-catering in spite of any financial hardships they might have to bear, as they expected, not only overeating, but also takeaways.

Ester: To be honest, I hosted my son’s first birthday and I had to take loan for it and it took me three years to pay it back! Like these affairs are expensive! We had to invite several families. We have to cater for everyone who attends and also for their takeaways for people who didn’t!

Participants shared similar dynamics and, especially, a main focus on food when entertaining guests at home, which also led to both overeating and unhealthy eating. The pressure to provide “good meals” in abundance, was so strong that some participants shared that, despite financial hardships and/or health concerns, they would still do their best to cook meat and a variety of dishes for visitors.

Kesa: Like we end up cooking more meat than usual during family visits. This may mean that we don’t have enough for the rest of the week or till the next shopping. But yes, we try to do our best to meet that expectation.

Sala: Yes, like you know how we alternate between veggies and meat or fish every day? And we enjoy simple meals, generally boiled leafy greens? All that goes out the window when we have people over [laughs]. You have to make something special and maybe a few types of dishes with meat and generally more rich food like add *lolo* (coconut cream) to the dishes. Like if we have a *lovo*, it’s a lot of meat, a lot of coconut cream. So, the meal does become very unhealthy. In fact, the meal becomes exactly what I discourage at home.

#### 3.1.2. Pressures Experienced by the Individual(s) Being Offered the Food

Mothers shared two main cultural norms that place pressure on the person being offered the food to consume it all, which often leads to unhealthy eat and overeating.

##### It’s Rude to Decline Food

Participants who had attended social gatherings shared that even when they felt full, they struggled with declining food that is offered due to fears of appearing disrespectful and/or unappreciative of all the time, effort, and money that the hosts had put in to prepare the feast. Participants who were on a special diet or were more conscious of how much they ate also shared that it was difficult to stick to their diets during social gatherings.

Ester: Also for us, in our culture, if anyone offers food, it’s kind of disrespectful to decline. Like it’s a bit rude and times you feel that when you decline you are giving the message that the food isn’t good either. So, it both sides [sic] and ends with people eating way more than they should.

Maggie: And if you are going in [a social gathering], there is a pressure that you cannot refuse something that they offer, like it’s disrespectful to do so. So much time, money, and effort is spent on preparing these feasts and it’s rude to then decline the food. And they are your family. It’s very difficult at times. Because you don’t want to appear that you are some high shot who is on a particular diet.

Leba: And then there comes someone saying, “You know your grandmother cooked that, you know, she put a lot of effort!” And then you feel obligated to have more, it’s like emotional blackmail.

Sala: I will still take a bit out of respect for the host so that they don’t feel offended and they are satisfied that I am enjoying the food they have prepared for us.

##### It’s Bad to Waste Food

Another factor that resulted in overconsumption was the importance placed on plate clearing in order to avoid wastage of food and the effort and money that was spent in preparing the food. According to the participants, wastage of food was greatly frowned upon within the iTaukei culture. Four main expressions of overeating to avoid food wastage were recorded in this study: (1) mothers sharing that they overate despite being full to avoid wastage; (2) children being encouraged to finish all the food that is dished out to them; (3) mothers eating food left over from their child’s plate to avoid wastage despite being full, and (4) the practice of taking out food for the elder members or the head of the family (usually older men) in a separate dish, which can then no longer be eaten by any other family member, indirectly pressurizing the individuals to consume all of the food that was kept aside for them. The following reports demonstrate these scenarios.

Maggie: But then because I was really wanting to eat more, I mean I love subways, I went ahead and bought another one and after a few bites, I was full but because of the money that was spent, I don’t want to waste it, so I ate all of it. [laughs]

Leba: So, I encourage my children to finish their food because we work really hard to, you know, to put the meals on the table. And it almost feels sinful to waste it you know… when there are so many kids, kids starving in the world. Mostly when we get, get together in church in our Fijian [iTaukei] occasions where my children, you know, and they cannot finish their food and they bring it over to me, so I just don’t … I make use of it, like finish it. I have to be the mother and finish their food so it’s not wasted.

Tevi: But I always take out food for my parents and put it aside before the rest of us eat. And no one else can eat that food. [] If they leave food, then it gets wasted. We can’t eat that food, so they never waste it. They finish all of it.

#### 3.1.3. Gender-Specific Norms

Three main forms of gender norms and expectations that encourage eating (and overeating) were identified by the mothers.

##### Men and Boys Need to Eat More Because They Engage in Hard Labor

All the participants in this study shared that men and boys were encouraged to eat more in the iTaukei culture, and the participants suggested that this was because men were engaged in more laborious tasks in comparison to women. Men were also seen as the heads of the household and, therefore, were offered more food.

Luisa: Men eat more than women because they do much of the hard work …, like work on the farm, harvesting, getting firewood. So, he eats more than any of us. And we have to make sure he eats well so that he can do all the hard work.

Kala: Men are generally encouraged to eat more, because men are heads of family, they sort of take the top place and they are expected to do hard work.

However, there also seemed to be a recognition among some mothers that the emphasis on hard labor was not always warranted, especially for their husbands and male family members who had more sedentary lifestyles. According to these mothers, the practice of offering more food to male family members was a norm in the iTaukei culture usually upheld by the elders in the family (generally women) who encouraged men and boys to eat more.

Mere: Oh yeah Shaz, like when we have my mother-in-law around, that’s what she does. “*Kana levu*… *Kana kana*” Like telling my husband and my son to eat more, more, more. Like you know in iTaukei, everyone eats like mountains. [Laughs]. Like for me, whatever, I take out. I know it’s enough for them.

Kesa: Yes. Yes, very much, even at home, in our family, my mother just makes sure of that, you know, my husband is offered food first at the table or if he comes home late, “Make sure his food is set aside, specifically for him.” If he goes outside for gardening, she would tell “Offer him some cassava, some tea.” So, culture still very much prevalent that way [Laughs].

Pressure on Expectant and Breastfeeding Mothers to “Eat for Two”

According to participants, pregnant and breastfeeding mothers are also encouraged to overeat for the health and wellbeing of the unborn child/baby. However, the mothers accepted that once they had weaned off their babies, they were told to reduce the amount of food they consumed and lose weight, which they struggled with.

Rusila: Yes, I remember when, when I got pregnant, in my early months of pregnancy I was still skinny, and oh my elders were telling me you’re not healthy the baby is suffering, you need to eat a lot, and I’m thinking, “What? What does that have to do with the baby?” [Laughs] I guess there’s some traditional and cultural tags to how much a male or female should eat, also if you are a breastfeeding mother, or a pregnant woman you are expected to eat a lot, compared to just a young teenager, they won’t expect you to eat a lot. [] I when I was pregnant with my daughter. They like, “Eat, eat, eat!” and then when I was breastfeeding, “Eat, eat, eat!” and after I had my daughter and I weaned off my daughter, they started “Stop eating, stop eating, stop eating!”

##### Bigger Body Sizes of Family Members Represent a Successful Mother or Wife

All the mothers in this study were actively involved in family meal planning and preparation, and many of the mothers also dished out the food for their families, especially for their children. One of the societal pressures that these women shared was how much society judged the success of a wife or mother based on the body sizes of their husbands and/or children. According to the women, one of the ways in which they found their community gauged how well she was performing her duties as a wife and mother was based on how her husband and children looked. Mothers and wives whose family members were deemed skinny or had recently lost weight were often branded as not performing their responsibilities of feeding their family. While some mothers also shared that the emphasis on bigger body sizes was shifting or that they now acknowledge that it is unhealthy to have bigger body sizes, all had felt this pressure of trying to feed their family members ‘appropriately’ to avoid criticism.

**Kesa:** And you know also, after marriage, my husband gained so much weight, and his family look at it as … that, that I am looking after him really well. That I am a good wife, and that used to make me feel good about myself. But we now know that it’s not healthy for him to be like that.

**Faith:** By looking at your family members, like they’re healthy and the way they grow, not skinny and all. So, so you’ve got a skinny kid, they think that you’re not doing so well as a mother. [] Um, they’ll say bad things about that mother. They’ll say, “she just go partying on, going here going there without taking care of the kids”. Like not giving them the right food, just giving them junk food. They always judge like that.

**Ester:** Yes, definitely. Oh, I get this a lot! Sometimes when you go to a function, people will be commenting “Hey, saying my son’s name, you so skinny! Your mum feeding you well?” It always makes me think, “Whether I am feeding my kids well? Whether I am a good mum?” I mean, I know they are not underweight. They are healthy weight, but you still get those comments. Especially before when I was 105 kgs, I would get comments like “Oh we know where all the food is going! It’s all going to mummy, mummy’s eating your share too” It used to really *really* affect me, I used to feel really down. But now I know that they are eating healthy and that matters more than them being chubby! [] Well for men, if someone says that my husband has lost weight, then there’s that hint or suggestion that I am not looking after my husband properly or that he is sick. So, in my experience, it’s the opposite for men. Gosh, there are so many mixed messages!

### 3.2. Misplaced Valuing of Foods that Cause Nutrition Transition and Unhealthy Eating

The mothers in this study also shared some cultural perceptions of food, especially in relation to the types of food that are valued within their communities. The misplaced value placed on these foods inevitably contribute to a nutrition transition and unhealthy eating.

#### 3.2.1. Meat Is for the Rich and Vegetables Are for the Poor

All participants shared that meat is, generally, highly desired and valued within iTaukei communities and is symbolic of a family’s affluence and status. Even participants who shared that they strove to include more vegetables in their diet were pressured into making more meat when entertaining or when celebrating. Serving meat for visitors was seen as a demonstration of the affluence of the family, and hosts would go to great lengths to live up to the image.

Kala: I think, from experience, if you go to a Fijian function or event and there’s no meat or not enough meat, people will talk about it. It’s just culture, it’s always how it’s been. And people look forward to a function for the food that’s going to be there, especially the pork, the beef, the chicken that’s going to be there. Our events revolve around our food and the meat is the main attraction.

Ester: So, if my family is eating healthy, like we are including more vegetables in our diet. I’m going to put salad on the table, include veggies in the soup but in these communal functions, we’ll see a big pig on the main table. And when people enter, that just shows how wealthy the host family is. But putting salads, adding veggies, it shows “Oh so this is how much they have in their bank account!” [Laughs]. Meat represents how wealthy a person is in iTaukei culture.

Kesa: Society does have an impact on the family and they are always judging, how the family is, what they eat, how they look, you know. [] And so, when they come, when they come home, they look at you know what’s being served. They look at the level of income and the level of, you know, what my husband and I are willing to do for whoever’s coming home, or the meal itself, you know, so I think sometimes the food that you eat defines the status of your life, I would say, your class. And that really affects the family, like trying to maintain that image. We have to try to serve meat when people visit because they will talk if we don’t.

One of the participants also shared that this concept of meat being associated with wealth whereas vegetables are for the poor is so ingrained within the culture that even young children make judgments on a family’s affluence based on the food that is served.

Sala: I want to share an incident. So, one of my nieces went to stay with a family for a few days and this family is of SDA [Seventh Day Adventist] faith, so you know they hardly eat meat. Anyways, when she came back, she told her parents that “We should help that family because they are very poor, they don’t get meat to eat. We should buy groceries for them”. You know what I mean, we need to change that mindset. That vegetables are for poor people only. For me, when I heard that, I realized that not only adults, but children also feel that, and it is so ingrained in our culture that meat is for the wealthy and vegetables are for poor people.

Families from lower socioeconomic backgrounds who could not afford meat looked for cheaper processed meat options, such as canned meat and fish and sausages, which they also indicated were easier to prepare.

Maggie: Ok, so sausages and tuna or egg sandwiches are easier lunches to prepare, but for me, the main thing is that … to be honest, I am a bit miser when it comes to buying chicken. When I do shopping, I hardly buy the chicken because, for me, like you buy one chicken and you cook it one day and it finishes but you can by 4–5 cans of tuna with the same money and it will last you a few meals. So, it’s really the price. I always opt for tuna, tinned fish, or sausages because you can spread it to a few meals.

Mel: I will do tinned fish and tuna, and corned mutton and, especially, sausages because we can’t afford fresh beef and pork.

#### 3.2.2. Energy Dense Foods Are More Satiating

Mothers also shared that satiation was an important consideration when planning meals, whereby they worried about what meals would be filling enough for the family members, especially men and children, to get them through to the next meal. To this end, mothers would encourage their children to eat more for dinner and would serve more energy dense foods for dinner, which was widely believed to be more “heavy” or satiating.

Kesa: And for my husband, on the days he has to go out of Suva, I make sure he eats his dinner well, like I might boil extra cassava or dalo [root crops] so that he has heavy dinner and that’s a heavy food. And at times the leftover cassava, he will have it for breakfast. And that keeps him going till lunch.

Mel: The cassava, it’s heavy, even if you eat a little bit of curry and whole of cassava, it will take you through the night.

#### 3.2.3. A Palate for Junk Food and Fast Foods

Mothers in this study acknowledged the negative health impacts of consumption of ultra-processed foods, such as sugary sweets, biscuits, and salty snacks and fast foods, such as fish and chips, fried chicken and chips, and burgers, however, they also shared that they and/or their family members love, and even crave for, these foods. Many of the mothers acknowledged buying snacks and fast foods for their families during their grocery shopping or whenever they could afford it.

Sala: They like cream biscuits like Tymo, and the normal Twisties, Bongo, Doritos and we buy it during grocery shopping. They study late and they keep snacking on this, like when they are under stress and have assignments due.

Mere: My kids love the blue packet Twisties [extra-cheese-flavored snack] and whenever we have extra [money] so then we buy some when grocery shopping or they go and buy from the shops around here.

For working parents, fast foods were also regularly consumed as lunch, when home cooked meals were not possible such as when running late from work, and as comfort foods after stressful days. Fast-food restaurants were also popular choices for celebrations and family treats, and participants shared that they loved the food and the general ambience of these restaurants.

Kesa: Because his work, they normally move around, Suva, Navua and Nausori so fast, fast food, yeah, fish and chips along the way … [] And for me, I have, the famous Southern Cross [fast food place on campus] fish and chips for lunch.

Sala: On shopping days we get fish and chips for the girls, they enjoy it. Or when we go out as a family for dinner, maybe twice a month.

Ru: So, you can imagine when I come home after that meeting [stressful day at work]. Oh man, if, if, if I have a lot of energy, maybe I could go, come with my stuff in the taxi, drive-through McDonald’s and just have only spicy chicken and sprite. I need those

Mel: When running late from work, I can’t cook, then we opt for fast food and takeaways. But especially on birthdays, anniversary, so when we are celebrating, then we try to do something special and save up so that we can eat Mc Donald’s or go out for pizza. [] I think it’s taste of the food, but also the atmosphere is also nice and that’s why you want to go somewhere special and enjoy your special occasion.

Finally, consumption of fast foods was also associated with affluence or the buying-power of the family, as evidenced by the following report:

Mel: So, it’s only when we can afford it. I think people only eat out when they can afford it so they have the money, they are rich, they can afford to go out for burgers and chicken and chips, pizza and all those kinds of food … One of my close friends is always taking her kids to fast food places and posting pictures, and I’m thinking “Man she has the money for it!”

## 4. Discussion

This study explored the sociocultural factors that are shaping nutrition transitions and influencing overconsumption and unhealthy eating within an urban iTaukei community. The food gatekeepers interviewed for this study identified several cultural norms, expectations, and pressures that influence overeating and unhealthy eating which, likely, also contribute to and maintain the high obesity rates within this community.

Participants shared that food and the actions of offering and receiving food within this community carried special meanings for them. People offering the food, whether they were the host of a large social gathering or a few guests at home or a mother feeding her family, were all expected to do their best to (1) provide good food, i.e., food that was valued within their culture, and (2) serve enough food so that all those who were eating felt satiated. This offering of food and encouraging people to eat until they were replete was seen as an integral part of “iTaukei hospitality” and was viewed as a demonstration of love, affection, and care for the person being offered the food. Therefore, it was incumbent upon the person receiving the food to not only graciously accept the food but also finish all of it, due to fears of appearing rude by declining this symbolism of love and affection. Participants shared that declining food was disrespectful or was thought of as being ungrateful of all the time, effort, and money that was spent on making the food. Thus, even if they had a different preference for food or already felt satiated, participants shared that they felt compelled to overeat when served more food, and they, themselves also encouraged plate-clearing, especially with their children.

Similar symbolism of offering food as communicating love, care, and affection [32,33] and as a means of strengthening social bonds [34,35] has been reported by others. However, its potential role in encouraging overeating and unhealthy eating has mainly been studied in the context of obesity and unhealthy eating in children (e.g., [33,36]). Also, studies on motivations behind plate-clearing have been limited to attitudes around avoiding food wastage (e.g., [37]), and the social and emotional pressures related to plate-clearing have not been studied. Nonetheless, studies indicate that encouragement of plate-clearing in childhood is a predictor of plate-clearing tendencies in adulthood [38], and plate-clearing attitudes are associated with higher body weight, even when accounting for other demographic and behavioral factors [39]. This suggests that the importance placed on plate-clearing in and the norms around iTaukei hospitality may be contributors to obesity in this community.

Further symbolism of food that contributed to overeating and unhealthy eating was also found in this study. When eating in company, the abundance of food and the display of a variety of food was often viewed as an indication of the host’s affluence or buying-power. Consequently, hosts would go to great lengths and even face financial hardships to cater for their guests. Two ways of demonstrating abundance of food were to encourage guests to eat more by assurances of plentiful food and to encourage takeaways. In social settings, kana meda bula (eat to live) was commonly used to urge guests to relinquish any inhibitions, such as concerns for health and well-being, and to enjoy the company and the food. Moreover, declining food in these settings was considered rude and was often also considered pretentious behavior: people who declined food were regarded as a “high shot who is on a particular diet”. In the context of strong views against wastage of food, participants felt compelled to overeat when served more food in social settings. These norms and practices around social eating will, understandably, have a greater impact on people whose socializing is accompanied by food and eating.

Gendered norms and expectations further encouraged overeating and had a wider impact in this community. Similar to the findings reported by Singh et al., [23] in their study of rural iTaukei communities, the notion that men and boys need to eat more as they engage in hard labor is also prevalent in this study of urban iTaukei community members, even where physical activity was minimal. In addition, this study also recorded an overtone of patriarchal attitudes, whereby men are seen as the head of the household and, therefore, are offered more food, regardless of the level of physical activity they engage in. Another finding recorded in this study was that participants described the pressure they felt in regard to the body size of their partners or husbands and children. The participating women indicated that they often felt judged if their children or partner looked skinny or had lost weight. These women also reported that the success of their role as a mother feeding her family was usually gauged based on how “healthy” their children and husbands appeared, and weight gain was viewed as a sign of a happy marriage and a healthy child. The pressures that mothers in this community face in regard to body size of their family members may be one of the reasons for the high levels of obesity and overweight observed amongst iTaukei children and adult males [25,40]. Moreover, since childhood obesity is a predictor of adulthood obesity and morbidity [41,42], the overfeeding of children can further exacerbate the obesity epidemic and the rates of death due to NCDs in this community. The women also faced their own pressures to overeat, albeit that they had also often been criticized for putting on weight. A common myth recounted by the participants was that mothers need to eat for two during pregnancy and breastfeeding. Pregnant and lactating mothers were often encouraged to overeat, which may in part explain the high obesity rates among women of reproductive age in this community [25].

Another key contributor to both the obesity pandemic and nutrition transitions in this community was the misplaced value on fast foods, junk food, and meat. Mothers in this study acknowledged that fast foods, junk food, and meat were not healthy, and as reported in previous studies (e.g., [23]), they also admitted that these foods were regularly consumed and enjoyed by their families. However, aside from factors such as convenience, accessibility, and affordability that have also been reported elsewhere (e.g., [23,43]), this study highlights additional reasons for dietary transitions in this urban indigenous community.

As with offering and receiving food, different types of food also had attached meanings that impacted their consumption. Meat, especially, was associated with being rich and affluent, and participants shared that, as a result, everyone aspired to serve and eat meat. Economic development has increased people’s ability to buy meat in this community and those who could not afford fresh meat often opted for cheaper processed meat, which is high in fats (e.g., canned meat and sausages) which increase their health risks related to NCDs. While this trend of higher consumption of meat, including red meat and processed meat is well documented in low- and middle-income countries (e.g., [44,45,46]), a concerning finding of this study was that the opposite was true for vegetables. Participants reported that eating more plant-based diets was often equated to being poor and that families who could not afford to eat meat, ate vegetables instead. Thus, when having visitors, participants could not be seen to be eating a lot of vegetables because this would imply that they were poor. The negative connotations attached to eating vegetables may be one reason for the low consumption of fruits and vegetables in this population [25,40]. This suggestion is further supported by our finding of families who were reportedly compelled to shift to a plant-based diet due to loss of income during COVID19 lockdowns [6].

Highly-processed foods (e.g., sugary sweets, biscuits, salty snacks) were described as tasty. Mothers acknowledged that their families, especially their children, enjoyed or wanted snacks for study sessions and, therefore, were routinely included in grocery shopping. Fast food was also viewed as delicious and often used as a family treat. Fast-food restaurants were also preferred venues for family celebrations, outings, and treats, due to the reportedly pleasant ambience of these restaurants. Moreover, the ability to afford fast foods was also associated with affluence. Lastly, a further driver of the obesity pandemic in this community might be the overemphasis on energy-dense foods, such as root crops, for satiety, which were often viewed as “heavy” or filling and were believed to keep a person satiated for longer. To this end, mothers would encourage family members to eat more energy-dense foods for dinner so that they would not get hungry during the night.

### 4.1. Public Health Implications

It is clear from national reports (e.g., [25,40]) that rates of obesity, overweight, unhealthy lifestyles, and NCDs in iTaukei communities in Fiji are high and increasing. This suggests that current interventions are insufficient or have limited impact. The findings of this study indicate that iTaukei cultural beliefs, norms, and practices greatly influenced dietary practices of the participants. In doing so, these sociocultural factors may not only create, but also maintain, an obesogenic social environment that facilitates and encourages overeating, unhealthy eating, and nutrient transitions within this community. This study underscores the importance of obesity interventions in urban iTaukei communities to address these sociocultural influences. The findings of this study indicate that interventions within this community are specifically needed to address (1) cultural beliefs and practices that emphasize abundance of food and overeating, plate-clearing, and attitudes such as *kana meda bula* which normalize and reinforce overeating and unhealthy eating, (2) cultural myths and beliefs around the energy needs of men and pregnant and lactating women and around equating larger body size as a sign of health and happiness, and (3) the misplaced value on meat, fast foods, junk foods, and energy-dense foods. The contextual factors that impact dietary practices identified in this study are likely to influence the effectiveness of current interventions. Developing effective partnerships with community stakeholders, including religious and community leaders may be pivotal in changing mindsets, promoting healthy lifestyles, and strengthening obesity-related interventions in this community.

### 4.2. Limitations

This study has several limitations and strengths. This study used qualitative interviews for an in-depth exploration of the sociocultural influences within the urban iTaukei community and the findings of this study cannot be extrapolated beyond this community. Moreover, this study relied on participants’ self-reports and is open to social-desirability and other reporting biases. Future research should consider the triangulation of findings by including other methods of data collection, expanding the sample to other regions in Fiji and the Pacific, including rural and maritime, and increasing the sample size. In noting these limitations, nonetheless, the study recruited relevant participants (food gatekeepers) in sufficient numbers to ensure data saturation and drew upon established data analysis procedures. Also, while this study was undertaken during a time when COVID-19 mitigation measures, such as lockdowns, that also impacted eating behaviors were in place, these have been addressed in a previous paper [6].

## 5. Conclusions

Literature on factors that impact food consumption in PICs has mainly focused on factors related to individuals, the physical environment, and macro-economics, and the impact of social and cultural factors have been largely under acknowledged. This study expands our understanding of sociocultural factors that may impact obesity and nutrient transitions in this urban indigenous community. Specifically, the study identified the cultural norms, beliefs, expectations, and pressures that contribute to overeating, unhealthy eating, and nutrition transitions within this community. Although future research is needed to test the strength of association between these norms, expectations, and pressures and obesity, the study has identified putative opportunities for obesity prevention strategies beyond trade policies and individual lifestyle changes. These may also inform the development of contextualized health-messaging and other interventions that mitigate or reduce these sociocultural influences to target obesity within this community and, more broadly, in low- and medium-income Pacific countries.

## Data Availability

The data presented in this study are not publically available due to restrictions of the informed consent obtained for this study.
